# Normal-Appearing Salivary Gland Ultrasonography Identifies a Milder Phenotype of Primary Sjögren's Syndrome

**DOI:** 10.3389/fmed.2020.602354

**Published:** 2020-12-09

**Authors:** Sara Zandonella Callegher, Alen Zabotti, Ivan Giovannini, Elena Treppo, Luca Quartuccio, Salvatore De Vita

**Affiliations:** Rheumatology Clinic, Department of Medical Area, Azienda Sanitaria Universitaria Friuli Centrale, Udine, Italy

**Keywords:** ultrasoud, salivary gland, scoring system, primary Sjogren's syndrome, follow-up, managment

## Abstract

**Objective:** Salivary gland ultrasound (SGUS) is emerging as a valid tool in the management of primary Sjögren's syndrome (pSS). This study aimed to investigate whether pSS patients with normal-appearing or pathological SGUS findings showed different clinical, laboratory, and pathologic pSS-related features, and to compare the results by using two different SGUS scores.

**Methods:** Consecutive pSS patients, according to the ACR-EULAR classification criteria, were evaluated. Salivary glands were scored using the early 1992 score by De Vita et al. and the latest 2019 OMERACT score, both being semiquantitative 0–3 scoring systems focused on ultrasonographic parenchymal inhomogeneity (grades 0 and 1, normal-appearing; grades 2 and 3, pathological). The patients were then divided into two groups: “SGUS normal-appearing” if all the salivary glands had normal-appearing parenchyma (grade 0 or 1), or “SGUS pathological” if the grade was 2 or 3 in at least one salivary gland. The associations between SGUS and pSS-related clinical, laboratory, and pathological features were then investigated in the two groups.

**Results:** One hundred pSS patients were evaluated, the mean age (±SD) was 60.9 ± 12.0 years, and mean disease duration was 11.7 ± 7.2 years. Twenty-nine out of 100 (29%) patients were in the “SGUS normal-appearing” group and 71/100 (71%) were in the “SGUS pathological” group. A normal-appearing SGUS was significantly associated with the absence of anti-La/SSB antibodies (*p* < 0.001) and normal unstimulated salivary flow rate (*p* = 0.02) by both univariate and multivariate analyses. By univariate analysis, a normal-appearing SGUS was significantly associated also with the absence of rheumatoid factor (*p* = 0.002) and of serum monoclonal component (*p* = 0.003), ESSDAI < 5 (*p* = 0.03), and with a negative lip biopsy (*p* = 0.029). No associations were found with other items, including anti-Ro/SSA (*p* = 0.145), Schirmer's test (*p* = 0.793), ESSPRI (*p* = 0.47), and demographic data. No differences in these results were observed by using the two SGUS scoring systems.

**Conclusion:** The SGUS allowed the identification of different phenotypes of pSS, and different SGUS scores focused on salivary gland inhomogeneity may be effective to this end.

## Introduction

Primary Sjögren's syndrome (pSS) is an autoimmune and lymphoproliferative connective tissue disease characterized by lymphocytic infiltration and damage of the salivary and lacrimal glands, leading to dryness of the mouth and eyes, and by additional possible glandular and extra-glandular manifestations (1).

Diagnosis and classification of pSS rely on a combination of clinical, laboratory, pathological, and imaging features, and among these, the minor salivary gland biopsy (MSGB) and anti-Ro/SSA autoantibodies have an essential classifying role (1, 2). Despite its high diagnostic and prognostic value, the MSGB has some limitations (3–5) and might be refused by the patient (6).

To assist the clinician in the diagnosis and management of pSS, several studies over the past three decades highlighted the potential of salivary gland ultrasound (SGUS) in describing the morphology of salivary glands in pSS (7, 8). Despite the initial encouraging data (9), only in recent years did the interest for the SGUS in pSS become widespread (10). Parenchymal inhomogeneity is the most important sonographic feature discriminating pSS patients from controls (10), and it is the basis of most SGUS scoring systems, with the long-standing available one developed by De Vita et al. (9) based on the results of stepwise discriminant analysis, and the most recent one developed in 2019 by the Outcome Measures in Rheumatology Clinical Trials (OMERACT) task force (11). Despite its good diagnostic performance (12), SGUS is not yet included in pSS classification criteria. This is mainly due to the poor agreement regarding the definitions of elementary sonographic lesions and scoring systems (10, 13), the scant evidence of intra- and inter-rater reliability (10, 14), and the use of old pSS cohorts for validation of pSS classification criteria, when SGUS was not yet fully developed (2). However, recently significant progress in these fields has been made (11, 15) and important research projects in pSS, e.g., HarmonicSS (16) and NECESSITY (17), are currently investigating the usefulness of SGUS in pSS. Although with some limitations for the reasons mentioned above, several studies reported associations between sonographic parenchymal inhomogeneity and clinical, laboratory, and pathological pSS-related features, such as reduced salivary flow rate, presence of anti-Ro/SSA and/or anti-La/SSB antibody, positive lip biopsy, etc. (18–27).

This study was focused on the possible stratification of pSS phenotypes by SGUS in a well-characterized cohort of patients fulfilling the most recent classification criteria. The results were compared by using the long-standing available and the most recent SGUS scoring system in pSS, i.e., the extremes of a period lasting almost three decades, during which many SGUS scores have been proposed, with substantial similarities. A possible agreement between the two “extreme” scores could substantiate the results found by several groups in these years about the usefulness of the SGUS in pSS.

## Methods

### Patients

Consecutive pSS patients referred to the Clinic of Rheumatology, University Hospital of Udine, Italy, from January 2019 until February 2020 were evaluated. Inclusion criteria were as follows: (a) fulfillment of the American College of Rheumatology–European League Against Rheumatism (ACR-EULAR) classification criteria for pSS (2); (b) available data on in-house performed SGUS and objective and subjective evaluations of dryness. Concomitant immunosuppressive therapy was allowed.

All patients gave oral and written informed consent for all procedures, which were carried out in accordance with the Declaration of Helsinki and with the guidelines for good clinical practice. The study was conducted according to a protocol approved by the Regional Ethical Committee (CEUR-2017-Os-027-ASUIUD) (16).

### Clinical and Laboratory Data

The following data were collected from patients' medical charts: gender, date of birth, pSS duration, presence of anti-Ro/SSA and anti-La/SSB antibodies, rheumatoid factor (RF), serum monoclonal component, serum cryoglobulinemia, complement C3 and C4 levels, and previous MSGB (28). The MSGB was considered positive if the focus score was ≥1; if the number of minor salivary glands was < 4 and/or the glandular surface area was < 8 mm^2^, the MSGB was considered not evaluable (28).

The oral and ocular dryness were assessed by both subjective, i.e., Visual Analog Scale (VAS) 0–10 oral and VAS 0–10 ocular sicca, and objective evaluations, i.e., the unstimulated salivary flow rate and the Schirmer's *I* test, performed the same day of the SGUS, according to the recommended procedures (29, 30).

The EULAR Sjögren's Syndrome Disease Activity Index (ESSDAI) for pSS systemic activity (31) and the EULAR Sjögren's Syndrome Patient Reported Index (ESSPRI) for the severity of patients' symptoms (32) were evaluated at the time of the SGUS. The ESSDAI is a composite index that evaluates 12 organ-specific domains; for each domain, features of disease activity were classified into three or four levels according to their severity, and the total score ranges from 0 to 123 points (31). Low disease activity is considered if the ESSDAI score is <5; moderate activity is considered if the ESSDAI score is 5–13, and high activity is considered if the ESSDAI score is ≥14 (33). The ESSPRI is a patient-reported index; the final score is the mean (0–10) of three items: VAS 0–10 of dryness, musculoskeletal pain, and fatigue (32). Patient-acceptable symptom state (PASS), defined as an ESSPRI <5, is the value below which patients consider themselves well (33).

### Ultrasonographic Assessment of Major Salivary Glands

**Figure 1:**
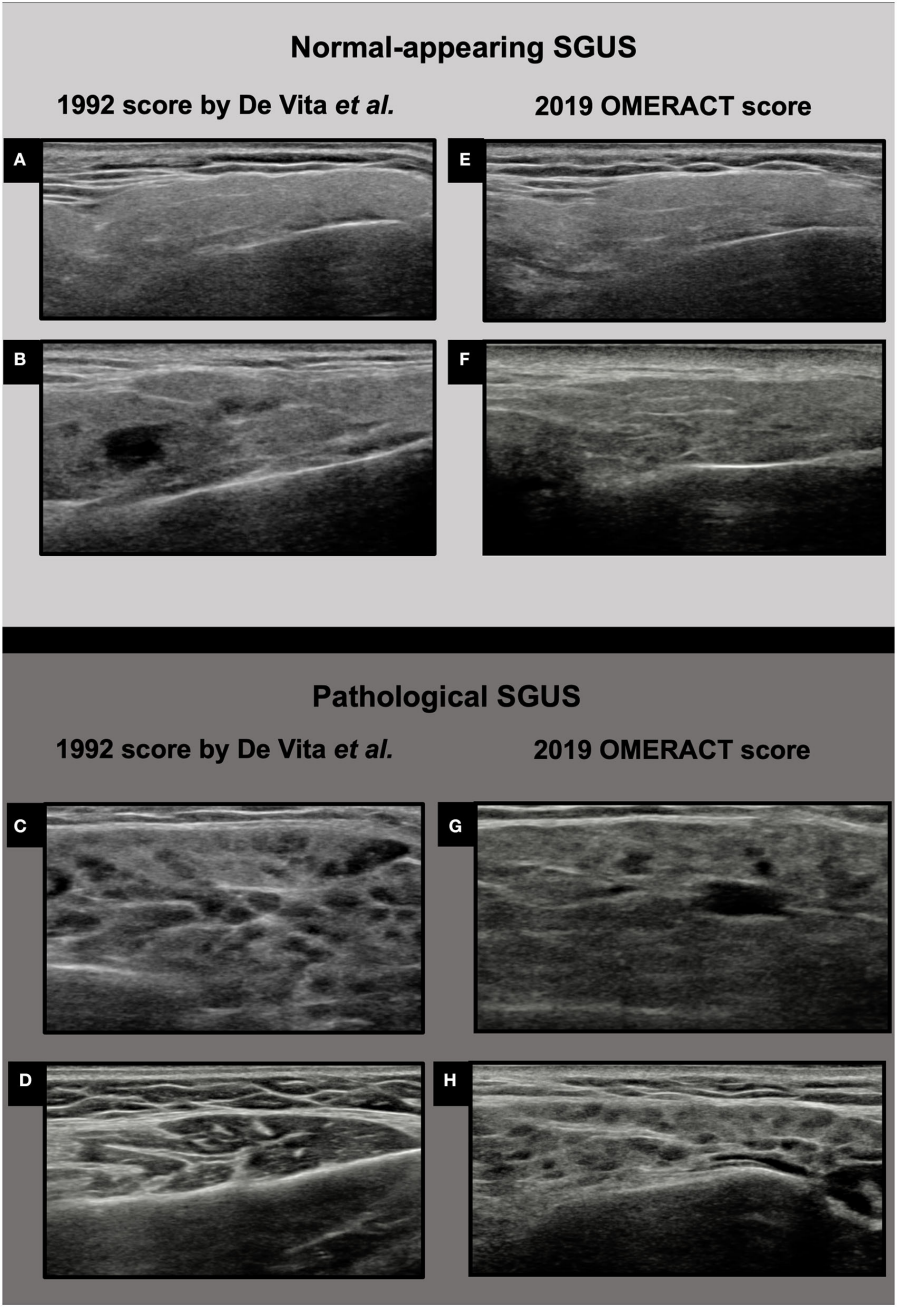
Ultrasound images of parotid glands in the two four-grade semiquantitative scoring systems. **(A)** De Vita et al. score grade 0; **(B)** De Vita et al. score grade 1; **(C)** De Vita et al. score grade 2; **(D)** De Vita et al. score grade 3; **(E)** OMERACT score grade 0; **(F)** OMERACT score grade 1; **(G)** OMERACT score grade 2; **(H)** OMERACT score grade 3.

The parotid glands (PGs) and the submandibular glands (SMGs) were examined using a SAMSUNG RS85 machine with a linear high-frequency transducer (LM4-15B) by one clinical investigator (AZ or SZC), blinded to clinical data of the patients. The intra-rater and inter-rater reliability between the two sonographers were >0.7 for both scores used. Both the PG and the SMGs were scanned with patients lying in the supine position with the neck hyper-extended and the head slightly turned to the opposite side. The PGs were evaluated in the longitudinal and cross-sectional plane and the SMGs were evaluated in the longitudinal plane, according to the OMERACT standardized scanning procedure (11). The US images were simultaneously scored at patient bedside using two four-grade semiquantitative scoring systems. The 1992 score by De Vita et al., developed by stepwise discriminant analysis, includes both anechoic/hypoechoic areas and hyperechoic bands (9). The four levels are as follows: grade 0, normal homogenous parenchyma; grade 1, mild inhomogeneity with isolated and small anechoic/hypoechoic areas without hyperechoic bands (definition updated in accordance with new ultrasound machines performance) (25, 34, 35); grade 2, moderate inhomogeneity with multiple anechoic/hypoechoic areas and/or few hyperechoic bands; grade 3, severe inhomogeneity with large and confluent anechoic/hypoechoic areas and/or diffuse hyperechoic bands (Figure 1). The OMERACT score is the most recent SGUS scoring system, and it is defined as follows: grade 0, normal parenchyma; grade 1, minimal change: mild inhomogeneity without anechoic/hypoechoic areas; grade 2, moderate change: moderate inhomogeneity with focal anechoic/hypoechoic areas but surrounded with normal tissue; grade 3, severe change: diffuse inhomogeneity with anechoic/hypoechoic areas occupying the entire gland surface (Figure 1) (11).

For each of the two SGUS scoring systems, for both the PG and the SMG, the worse finding of the two sides was used in the analyses. The scores were then dichotomized and converted into “normal-appearing,” if the original score was 0 or 1, and “pathological,” if the original score was 2 or 3, according to previous studies (9, 15, 21). The patients were divided into two groups according to the SGUS results: “SGUS normal-appearing group,” in which the patients had both the PG and the SMG with normal-appearing glandular parenchyma (grade 0 or 1 in both PG and SMG), and the “SGUS pathological group,” in which the patients had at least one between PG and SMG with a pathological score (grade 2 or 3 in at least one between PG and SMG).

### Statistical Analyses

Categorical variables are presented as frequencies and percentages. Quantitative variables are described with the mean ± standard deviation (SD), or with the median (range), as appropriate.

Chi-square test or Fisher exact test was applied to assess differences for categorical variables, while *t* test or Mann–Whitney test was applied to assess differences for continuous variables. All significance tests were two-tailed, and results with *p* < 0.05 were considered statistically significant. The odds ratio (OR) was expressed with 95% CI.

In order to assess which clinical or laboratory data were independently associated with a normal SGUS score, a multivariate logistic regression analysis was used, which included all the variables showing a significance *p* < 0.05 at the univariate analysis and which were available in all the patients.

## Results

### Clinical and Laboratory Characteristics of All Cohorts

One hundred consecutive pSS patients were evaluated, 92/100 (92%) were females, the mean age (±SD) at the SGUS evaluation was 60.9 ± 12.0 years, and the mean disease duration was 11.7 ± 7.2 years. Patients positive for anti-Ro/SSA were 95/100 (95%), while patients positive for anti-La/SSB were 52/100 (52%); all patients positive for anti-La/SSB are also positive for anti-Ro/SSA. RF was positive in 59/100 (59%) patients. An abnormal unstimulated salivary flow rate was found in 71/100 (71%) patients, while an abnormal Schirmer's *I* test was found in 78/100 (78%). The MSGB data were available in 49/100 (49%) patients, with a positive result in 37/49 (75.5%); in most patients, the MSGB was performed >5 years before the SGUS, at the time of pSS diagnosis. The median (range) ESSDAI at the time of the SGUS evaluation was 3 (0–22), while mean (±SD) ESSPRI was 5.7 ± 2. Detailed clinical and laboratory data are reported in Table 1.

**Table 1:** Patients' demographic, clinical, and laboratory features.

	**All patients**	**SGUS normal-appearing group**	**SGUS pathological group**	***p-*Value normal vs. pathological**	**Odds ratio (95% CI)**
**Demographic data**					
Number of patients	100	29	71		
Gender, female, *n* (%)	92/100 (92%)	25/29 (86.2%)	67/71 (94.4%)	0.225	
Age at disease onset, years, *mean ± SD; median*	49.3 ± 12.1; 49.5	49.2 ± 12.2; 49	49.3 ± 12.2; 50	0.980	
Age at evaluation, years, *mean ± SD; median*	60.9 ± 12.0; 61	59.9 ± 11.6; 58	61.4 ± 12.2; 62	0.591	
Disease duration, years, *mean ± SD; median*	11.7 ± 7.2; 10.5	10.7 ± 6.6; 9	12.0 ± 7.5; 12	0.482	
**Serological features**					
Anti-Ro/SSA positive, *n* (%)	95/100 (95%)	26/29 (89.6%)	69/71 (97.2%)	0.145	
Anti-La/SSB positive, *n* (%)	52/100 (52%)	8/29 (27.6%)	44/71 (62.0%)	**0.002**	4.3 (1.7–11)
Rheumatoid factor positive, *n* (%)	59/100 (59%)	10/29 (34.5%)	49/71 (69.0%)	**0.002**	4.2 (1.7–10.6)
Presence of serum monoclonal component, *n* (%)	28/100 (28%)	2/29 (6.9%)	26/71 (36.6%)	**0.003**	7.8 (1.7–35.5)
Low complement C3 and/or C4 level, *n* (%)	24/100 (24%)	5/29 (17.2%)	19/71 (26.8%)	0.44	
Presence of serum cryoglobulinemia, *n* (%)	13/100 (13%)	1/29 (3.4%)	12/71 (16.9%)	0.10	
**Clinical features**					
Lip biopsy focus score ≥1, *n* (%)	37/49 (75.5%)	8/15 (53.3%)	29/34 (85.3%)	**0.029**	5.1 (1.3–20.3)
Abnormal unstimulated salivary flow rate^§^, *n* (%)	71/100 (71%)	13/29 (44.8%)	58/71 (81.7%)	** <0.001**	5.5 (2.1–14.2)
Abnormal Schirmer's *I* test^*^, *n* (%)	78/100 (78%)	22/29 (75.9%)	56/71 (78.9%)	0.793	
ESSDAI, *median (range)*	3 (0–22)	2 (0–18)	4 (0–22)	0.078	
ESSDAI <5, *n* (%)	66/100 (66%)	24/29 (82.7%)	42/71 (59.1%)	**0.03**	3.3 (1.1–9.7)
ESSPRI, *mean ± SD; median*	5.7 ± 2.1; 6	5.5 ± 2.1; 5.7	5.8 ± 2.1; 6	0.470	
PASS, ESSPRI <5, *n* (%)	36/100 (36%)	11/29 (37.9%)	25/71 (35.2%)	0.821	
VAS oral dryness, *mean ± SD; median*	6.7 ± 2.7; 7	6 ± 2.3; 6	7 ± 2.8; 7	0.08	
VAS ocular dryness, *mean ± SD; median*	6.0 ± 2.6; 6	5.9 ± 1.5; 6	6.1 ± 2.9; 7	0.525	

§ *Unstimulated salivary flow rate: ≤ 1.5 mL/15 min was considered pathological*.

* *Schirmer's I test values <5 mm/5 min were considered pathological*.

*95% CI, confidence interval; ESSDAI, EULAR Sjögren's Syndrome Disease Activity Index; ESSPRI, EULAR Sjögren's Syndrome Patient Reported Index; PASS, patient acceptable symptom state; SGUS, salivary gland ultrasound; VAS, Visual Analog Scale*.

*Bold values are the statistic significant p-value (p < 0.05)*.

### Sonographic Evaluation and Patients' Stratification

The results of the two four-grade SGUS scoring systems were similar and are shown in Table 2. The dichotomized OMERACT score for the PG was normal-appearing in 37/100 (37%) and pathological in 63/100 (63%) patients, while that for the SMG was normal-appearing in 39/100 (39%) and pathological in 61/100 (61%) patients. The dichotomized score by De Vita et al. for the PG was normal-appearing in 32/100 (32%) and pathological in 68/100 (68%) patients, while for the SMG, it was normal-appearing in 35/100 (35%) and pathological in 65/100 (65%) patients.

**Table 2:** Sonographic features. Number of patients (%) for each grade of the two semiquantitative scoring systems OMERACT and De Vita et al. score for parotid gland and submandibular glands.

		**OMERACT score** ***n*** **=** **100**		**Score by De Vita et al**.***n*** **=** **100**	
		**PG, *n* (%)**	**SMG, *n* (%)**	**PG, *n* (%)**	**SMG, *n* (%)**
Semiquantitative score 0–3	Grade 0	20 (20%)	19 (19%)	22 (22%)	21 (21%)
	Grade 1	17 (17%)	20 (20%)	10 (10%)	14 (14%)
	Grade 2	37 (37%)	43 (43%)	40 (40%)	28 (28%)
	Grade 3	26 (26%)	18 (18%)	28 (28%)	37 (37%)
Normal-appearing score vs.pathological score	Normal-appearing (grade 0 or 1)	37 (37%)	39 (39%)	32 (32%)	35 (35%)
	Pathological (grade 2 or 3)	63 (63%)	61 (61%)	68 (68%)	65 (65%)
Normal-appearing SGUS vs. pathological SGUS	Normal-appearing (grade 0 or 1 in both PG and SMG)	29 (29%)		29 (29%)	
	Pathological (grade 2 or 3 in at least one between PG or SMG)	71 (71%)		71 (71%)	

*In the second part of the table, the results of the dichotomized score (normal-appearing vs. pathological) for both OMERACT score and the score by De Vita et al*.

*PG, parotid gland; SGUS, salivary gland ultrasound; SMG, submandibular gland*.

The pSS patients were then divided into two groups according to these SGUS results: 29/100 (29%) patients had a normal-appearing SGUS in both the PG and SMG (grade 0 or 1 in both), and were then assigned to the “SGUS normal-appearing group,” while 71/100 (71%) patients had a pathological SGUS in at least one PG or SMG (grade 2 or 3 in at least one between PG and SMG) and were then assigned to the “SGUS pathological group.” The division was the same for both the 1992 score and the 2019 OMERACT score (Table 2).

### Clinical and Laboratory Features Associated With Normal-Appearing SGUS

The clinical and laboratory features of the two groups of patients are reported in Table 1.

#### Univariate Analysis

**Figure 2:**
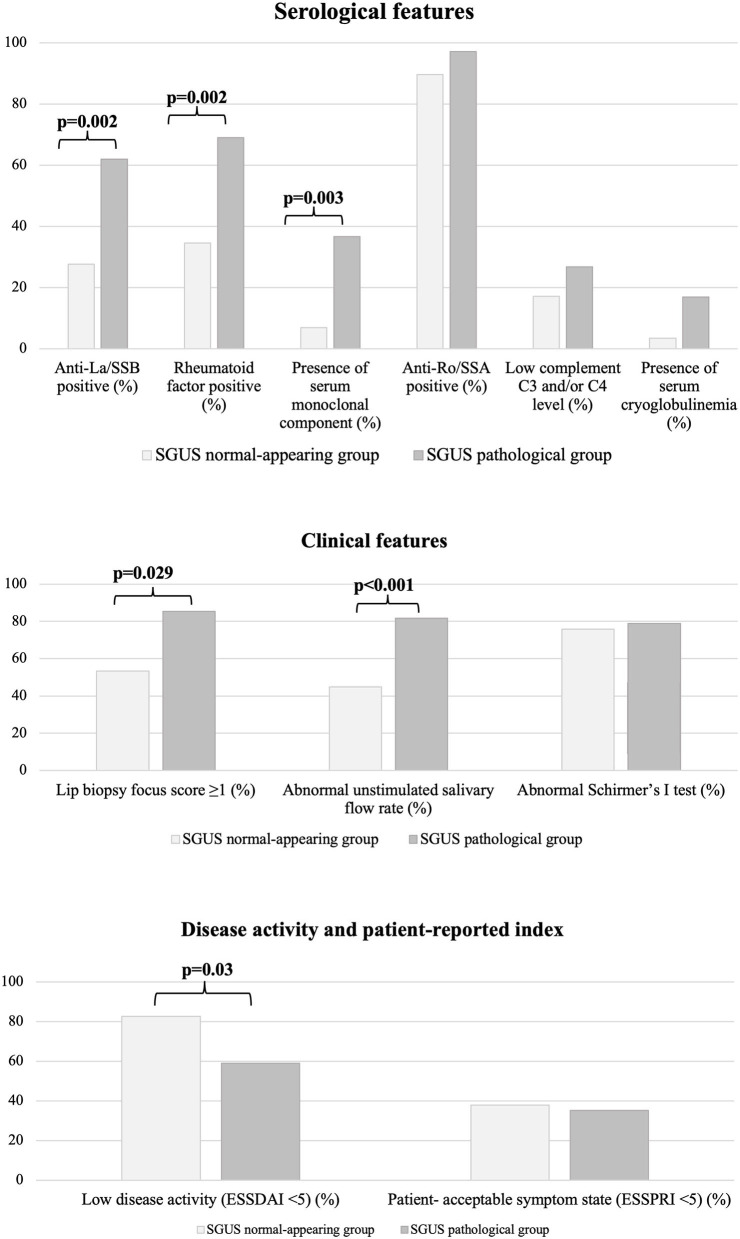
Comparison of serological and clinical features among the two groups of patients.

By univariate analysis, a normal-appearing SGUS was significantly associated with the absence of anti-La/SSB antibodies [8/29 (27.6%) vs. 44/71 (62%), *p* = 0.002, OR 4.3 (95% CI 1.7–11)], RF [10/29 (34.5%) vs. 49/71 (69%), *p* = 0.002, OR 4.2 (95% CI 1.7–10.6)], and serum monoclonal component [2/29 (6.9%) vs. 26/71 (36.6%), *p* = 0.003, OR 7.8 (95% CI (1.7–35.5)]. A normal-appearing SGUS was also associated with a normal unstimulated salivary flow rate [13/29 (44.8%) vs. 58/71 (81.7%), *p* < 0.001, OR 5.5 (95% CI 2.1–14.2)] and with a negative MSGB [8/15 (53.3%) vs. 29/34 (85.3%), *p* = 0.029, OR 5.1 (95% CI 1.3–20.3)] (Figure 2).

Finally, a normal-appearing SGUS was associated with a low disease activity [ESSDAI < 5, 24/29 (82.7%) vs. 42/71 (59.1%), *p* = 0.03, OR 3.3 (95% CI 1.1–9.7)] (Figure 2). In detail, patients with a normal-appearing SGUS had lower activity in the glandular (*p* = 0.033) and in the biological (*p* = 0.048) ESSDAI domains, while no differences were found in the other domains (Supplementary Table 1). No associations were found between normal-appearing SGUS and other serological, clinical, or demographic data (Table 1).

#### Multivariate Analysis

By multivariate analysis, a normal-appearing SGUS was significantly associated with the absence of anti-La/SSB antibodies [*p* < 0.001, OR 9.0 (95% CI 2.6–30.8)] and normal unstimulated salivary flow rate [*p* = 0.02, OR 3.8 (95% CI 1.2–11.8)] (Table 3).

**Table 3:** Clinical and serological features associated with normal-appearing SGUS by multivariate analysis.

**Variable**	**OR**	**95% CI**	***p-*Value**
Positive anti-La/SSB	9.025	2.647–30.771	** <0.001**
Positive rheumatoid factor	0.717	0.208–2.471	0.599
Presence of serum monoclonal component	3.2	0.72–14.214	0.126
Abnormal unstimulated salivary flow rate	3.785	1.21–11.84	**0.022**
ESSDAI <5	3.103	0.772–12.474	0.111

*95% CI, confidence interval; ESSDAI, EULAR Sjögren's Syndrome Disease Activity Index; OR, odds ratio*.

*Bold values are the statistic significant p-value (p < 0.05)*.

### Agreement Between the SGUS and the MSGB

The MSGB data were available in 49/100 (49%) patients. The agreement between the SGUS and the MSGB was moderate (73.5%, 36/49) (Supplementary Figure 1). In patients with a positive MSGB (37/49, 75.5%), the SGUS was pathological as well in 29/37 (78.4%). However, the SGUS was positive also in 5/12 (41.7%) pSS patients with a negative MSGB, all being anti-SSA-positive. Overall, among the 49 pSS patients who performed both MSGB and SGUS, there were 8/49 (16.3%) who were positive only by MSGB, and 5/49 (10.2%) only by SGUS. Among the five pSS patients being anti-SSA-negative/MSGB-positive, the SGUS was positive in 2/5 (40%).

## Discussion

This study supported the clinical usefulness of the SGUS in the management of pSS since it allowed to identify distinct pSS phenotypes with different outcomes. In this study indeed, a normal-appearing SGUS was significantly associated with low disease activity (ESSDAI <5), less salivary gland function impairment (i.e., normal unstimulated salivary flow rate), negative minor salivary gland histopathology, and the lack of RF, anti-La/SSB antibodies, and serum monoclonal component. Thus, pSS patients with a normal-appearing SGUS may represent a clinical phenotype with milder disease and probably a lower lymphoma risk based on present knowledge (36–40). Improving the identification of a milder disease subset is an unmet need in pSS (41), and therefore, the SGUS could be of major value to this end. Moreover, the SGUS may be employed to detect and to guide diagnostic biopsy of salivary gland lymphoma (42, 43), which is one of the main causes of increased mortality in pSS (36), and to follow-up pSS patients with parotid swelling, i.e., a major risk factor or an early manifestation of lymphoma itself (44).

Since we noticed only slight differences in the sonographic elementary lesions considered in the several SGUS scoring systems employed up to now, all mainly focused on parenchymal inhomogeneity (9, 11, 21, 45–47), we decided to use the two “extreme” scores of the three decades of application of the SGUS in pSS, both easy to apply and little time consuming (9, 11). The early one was proposed by our group in 1992 (9) and is based on parenchymal inhomogeneity, which was selected by multivariate analysis as the SGUS variable that best identified pSS patients. The latest was proposed in 2019 by the OMERACT task force and is also focused on parenchymal inhomogeneity (11). Of note, two different SGUS scoring systems provided identical results in this study, and this might be relevant to facilitate, at least in part, the evaluation and the comparison of the previous studies which used different scoring systems (18–27), although in our study, a bias in classifying the same US image simultaneously at patient bedside using two different, but quite similar, scoring systems could be present.

Unlike other studies (18–25), we evaluated only patients who fulfilled the latest ACR-EULAR classification criteria, while patients suffering from non-pSS sicca syndrome were excluded from evaluation.

In this study, confirming the results of Theander and Mandl (21), patients with either normal-appearing or pathological SGUS were not different for age or disease duration (more than 10 years for both subgroups), indicating that the SGUS abnormalities appeared to be more likely linked to patients' intrinsic characteristics and disease severity, rather than being secondary to age or disease duration (48). Moreover, Baldini and colleagues reported that changes in the salivary gland parenchymal echostructure developed relatively early in the disease course ( ≤ 5 years from the onset of symptoms) (49). Further prospective studies are needed to assess the differences between the sonographic pattern of pSS patients with early or long-standing disease, evaluating in depth the sonographic elementary lesions (13, 34).

Unlike Hammenfors and colleagues (18) in the present study, the SGUS findings were not associated with subjective and objective lacrimal impairment. A possible uncoupling between the severity of oral and ocular involvement is still little recognized in pSS, and the use of ultrasonography may be helpful to further disclose it (50), then additional researches are needed.

Of note, SGUS has proven to be sensitive to change after treatment (51–53) and to be able to identify active inflammatory lesions, the anechoic/hypoechoic areas, and the damage-related lesions, the hyperechoic bands, i.e., those sonographic lesions mainly associated with salivary impairment (34), with researching and therapeutic implications. The relevance of a clear distinction between activity-related and damage-related features was recently highlighted by experts within the HarmonicSS cooperative research project (16), and also a subsequent cooperative project in pSS, named NECESSITY, is currently exploring the use of the SGUS in pSS (17).

A final key issue is the relationship between the SGUS and the minor salivary gland pathology in pSS. The histopathology of the major salivary glands may in part differ from the findings in the MSGB in pSS patients, and only one group approached this issue up to now (54, 55). The major limitations of our study are that MSGB was available in only one-half of the patients, and that almost all patients (95%) were anti-Ro/SSA positive, so it was not possible to evaluate the association between the SGUS pattern and the presence of anti-Ro/SSA antibodies. In these 49 pSS patients, the agreement between the SGUS and the MSGB was substantial (73.5%), but the SGUS resulted positive also in a fraction (5/12, 41.7%) of MSGB-negative patients. On the other hand, SGUS was positive in 2/5 (40%) anti-SSA-negative/MSGB-positive patients. Therefore, anti-SSA/SSB antibody test, MSGB, and SGUS might represent complementary tools in pSS.

In conclusion, this study highlighted that the SGUS may improve pSS stratification in clinically different phenotypes, and therefore, it may be usefully integrated in the clinical management of pSS patients. Salivary gland parenchymal inhomogeneity is confirmed as the key SGUS abnormality in pSS.

## Data Availability Statement

The raw data supporting the conclusions of this article will be made available by the authors, without undue reservation.

## Ethics Statement

The studies involving human participants were reviewed and approved by Regional Ethical Committee (CEUR-2017-Os-027-ASUIUD). The patients/participants provided their written informed consent to participate in this study.

## Author Contributions

AZ, SZ, and SDV contributed to study conception and design. AZ, SZ, LQ, and SDV contributed to analysis and interpretation of data. All authors contributed to acquisition of data and to draft the article or to revise it critically for important intellectual content. All authors agreed for all aspects of the work in ensuring that questions related to the accuracy or integrity of any part of the work were appropriately investigated and resolved. All authors approved the final version of the article to be published.

## Conflict of Interest

The authors declare that the research was conducted in the absence of any commercial or financial relationships that could be construed as a potential conflict of interest. The handling editor declared a past co-authorship with two of the authors LQ, SDV.

## Abbreviations

ACR-EULAR: American College of Rheumatology/European League Against Rheumatism
ESSDAI: EULAR Sjögren's Syndrome Disease Activity Index
ESSPRI: EULAR Sjögren's Syndrome Patient Reported Index
MSGB: minor salivary gland biopsy
OMERACT: Outcome Measures in Rheumatology Clinical Trials
PASS: patient-acceptable symptom state
PG: parotid gland
pSS: primary Sjögren's syndrome
RF: rheumatoid factor
SGUS: salivary gland ultrasound
SMG: submandibular gland
VAS: Visual Analog Scale.

## References

[1] Goules AV Tzioufas AG Primary Sjögren's syndrome: clinical phenotypes, outcome and the development of biomarkers. Immunol Res. (2017) 65:331–44. 10.1007/s12026-016-8844-4 27444892

[2] Shiboski CH Shiboski SC Seror R Criswell LA Labetoulle M Lietman TM. 2016 American College of Rheumatology/European League Against Rheumatism Classification Criteria for Primary Sjögren's Syndrome: a consensus and data-driven methodology involving three international patient cohorts: ACR/EULAR classification criteria for primary SS. Arthritis Rheumatol. (2017) 69:35–45. 10.1136/annrheumdis-2016-210571 27785888 PMC5650478

[3] Guellec D Cornec D Jousse-Joulin S Marhadour T Marcorelles P Pers J-O. Diagnostic value of labial minor salivary gland biopsy for Sjögren's syndrome: a systematic review. Autoimmun Rev. (2013) 12:416–20. 10.1016/j.autrev.2012.08.001 22889617

[4] Theander E Vasaitis L Baecklund E Nordmark G Warfvinge G Liedholm R. Lymphoid organisation in labial salivary gland biopsies is a possible predictor for the development of malignant lymphoma in primary Sjögren's syndrome. Ann Rheum Dis. (2011) 70:1363–8. 10.1136/ard.2010.144782 21715359 PMC3128323

[5] Carubbi F Alunno A Cipriani P Di Benedetto P Ruscitti P Berardicurti O. Is minor salivary gland biopsy more than a diagnostic tool in primary Sjögren?s syndrome? Association between clinical, histopathological, and molecular features: a retrospective study. Semin Arthritis Rheum. (2014) 44:314–24. 24935529 10.1016/j.semarthrit.2014.05.015

[6] Colella G Cannavale R Vicidomini A Itro A. Salivary gland biopsy: a comprehensive review of techniques and related complications. Rheumatology (Oxford). (2010) 49:2117–21. 10.1093/rheumatology/keq225 20660500

[7] Devauchelle-Pensec V Zabotti A Carvajal-Alegria G Filipovic N Jousse-Joulin S De Vita S. Salivary gland ultrasonography in primary Sjögren's syndrome: opportunities and challenges. Rheumatology. (2019) kez079. 10.1093/rheumatology/kez079 30892624

[8] Baldini C Zabotti A Filipovic N Vukicevic A Luciano N Ferro F. Imaging in primary Sjögren's syndrome: the “obsolete and the new”. Clin Exp Rheumatol. (2018) 36 (Suppl.) 112:215–21. 30156542

[9] De Vita S Lorenzon G Rossi G Sabella M Fossaluzza V. Salivary gland echography in primary and secondary Sjögren's syndrome. Clin Exp Rheumatol. (1992) 10:351–6. 1395220

[10] Jousse-Joulin S Milic V Jonsson MV Plagou A Theander E Luciano N. Is salivary gland ultrasonography a useful tool in Sjögren's syndrome? A systematic review. Rheumatology (Oxford). (2016) 55:789–800. 10.1093/rheumatology/kev385 26667216

[11] Jousse-Joulin S D'Agostino MA Nicolas C Naredo E Ohrndorf S Backhaus M. Video clip assessment of a salivary gland ultrasound scoring system in Sjögren's syndrome using consensual definitions: an OMERACT ultrasound working group reliability exercise. Ann Rheum Dis. (2019) 78:967–73. 10.1136/annrheumdis-2019-215024 31036626

[12] Delli K Dijkstra PU Stel AJ Bootsma H Vissink A Spijkervet FKL. Diagnostic properties of ultrasound of major salivary glands in Sjögren's syndrome: a meta-analysis. Oral Dis. (2015) 21:792–800. 10.1111/odi.12349 25988616

[13] Jousse-Joulin S Nowak E Cornec D Brown J Carr A Carotti M. Salivary gland ultrasound abnormalities in primary Sjögren's syndrome: consensual US-SG core items definition and reliability. RMD Open. (2017) 3:e000364. 10.1136/rmdopen-2016-000364 28879042 PMC5575597

[14] Fox RI. Is salivary gland ultrasonography a useful tool in Sjögren's syndrome? Rheumatology (Oxford). (2016) 55:773–4. 10.1093/rheumatology/kev409 26827269

[15] Jousse-Joulin S Gatineau F Baldini C Baer A Barone F Bootsma H. Weight of salivary gland ultrasonography compared to other items of the 2016 ACR/EULAR classification criteria for Primary Sjögren's syndrome. J Intern Med. (2020) 287:180–8. 10.1111/joim.12992 31618794

[16] HarmonicSS—HARMONIzation and integrative analysis of regional national and international Cohorts on primary Sjögren's Syndrome (pSS) towards improved stratification treatment and health policy making Available online at: https://www.harmonicss.eu/ (accessed November 23, 2020).

[17] NECESSITY—NECESSITY New clinical endpoints in primary Sjögren's syndrome: an interventional trial based on stratifying patients Available online at: https://www.necessity-h2020.eu/ (accessed November 24, 2020).

[18] Hammenfors DS Brun JG Jonsson R Jonsson MV. Diagnostic utility of major salivary gland ultrasonography in primary Sjögren's syndrome. Clin Exp Rheumatol. (2015) 33:56–62. 25535773

[19] Lee K-A Lee S-H Kim H-R. Diagnostic and predictive evaluation using salivary gland ultrasonography in primary Sjögren's syndrome. Clin Exp Rheumatol. (2018) 36(Suppl. 112):165–72. 29600950

[20] Fidelix T Czapkowski A Azjen S Andriolo A Trevisani VFM. Salivary gland ultrasonography as a predictor of clinical activity in Sjögren's syndrome. PLoS ONE. (2017) 12:e0182287. 10.1371/journal.pone.0182287 28783737 PMC5544350

[21] Theander E Mandl T. Primary Sjögren's syndrome: diagnostic and prognostic value of salivary gland ultrasonography using a simplified scoring system. Arthritis Care Res (Hoboken). (2014) 66:1102–7. 10.1002/acr.22264 24339361

[22] Nieto-González JC Ovalles-Bonilla JG Estrada E Serrano-Benavente B Martínez-Barrio J González-Fernández CM. Salivary gland ultrasound is linked to the autoimmunity profile in patients with primary Sjögren's syndrome. [published correction appears in *J Int Med Res*. (2018) 46:3991]. J Int Med Res. (2020) 48:300060518767031. 10.1177/0300060518767031 29644928 PMC7113485

[23] Inanc N Sahinkaya Y Mumcu G Türe Özdemir F Paksoy A Ertürk Z. Evaluation of salivary gland ultrasonography in primary Sjögren's syndrome: does it reflect clinical activity and outcome of the disease? Clin Exp Rheumatol. (2019) 37(Suppl. 118):140–5. 31287407

[24] Wernicke D Hess H Gromnica-Ihle E Krause A Schmidt WA. Ultrasonography of salivary glands—a highly specific imaging procedure for diagnosis of Sjögren's syndrome. J Rheumatol. (2008) 35:285–93. 18203316

[25] Milic V Colic J Cirkovic A Stanojlovic S Damjanov N. Disease activity and damage in patients with primary Sjogren's syndrome: prognostic value of salivary gland ultrasonography. PLoS ONE. (2019) 14:e0226498. 10.1371/journal.pone.0226498 31891590 PMC6938326

[26] Kim J-W Lee H Park S-H Kim S-K Choe J-Y Kim JK. Salivary gland ultrasonography findings are associated with clinical, histological, and serologic features of Sjögren's syndrome. Scand J Rheumatol. (2018) 47:303–10. 10.1080/03009742.2017.1374451 29411664

[27] La Paglia GMC Sanchez-Pernaute O Alunno A Martínez-Becerra MJ Romero-Bueno F Recuero S. Ultrasound salivary gland involvement in Sjogren's syndrome vs. other connective tissue diseases: is it autoantibody and gland dependent? Clin Rheumatol. (2020) 39:1207–15. 10.1007/s10067-019-04780-2 31676972

[28] Fisher BA Jonsson R Daniels T Bombardieri M Brown RM Morgan P. Standardisation of labial salivary gland histopathology in clinical trials in primary Sjögren's syndrome. Ann Rheum Dis. (2017) 76:1161–8. 10.1136/annrheumdis-2016-210448 27965259 PMC5530351

[29] Navazesh M Kumar SK University of Southern California School of Dentistry. Measuring salivary flow: challenges and opportunities. J Am Dent Assoc. (2008) 139(Suppl.):35S−40S. 10.14219/jada.archive.2008.0353 18460678

[30] Whitcher JP Shiboski CH Shiboski SC Heidenreich AM Kitagawa K Zhang S. A simplified quantitative method for assessing keratoconjunctivitis sicca from the Sjögren's Syndrome International Registry. Am J Ophthalmol. (2010) 149:405–15. 10.1016/j.ajo.2009.09.013 20035924 PMC3459675

[31] Seror R Ravaud P Bowman S Baron G Tzioufas A Theander E. EULAR Sjogren's syndrome disease activity index: development of a consensus systemic disease activity index for primary Sjogren's syndrome. Ann Rheum Dis. (2010) 69:1103–9. 10.1136/ard.2009.110619 19561361 PMC2937022

[32] Seror R Ravaud P Mariette X Bootsma H Theander E Hansen A. EULAR Sjögren's Syndrome Patient Reported Index (ESSPRI): development of a consensus patient index for primary Sjögren's syndrome. Ann Rheum Dis. (2011) 70:968–72. 10.1136/ard.2010.143743 21345815

[33] Seror R Bootsma H Saraux A Bowman SJ Theander E Brun JG. Defining disease activity states and clinically meaningful improvement in primary Sjögren's syndrome with EULAR primary Sjögren's syndrome disease activity (ESSDAI) and patient-reported indexes (ESSPRI). Ann Rheum Dis. (2016) 75:382–9. 10.1136/annrheumdis-2014-206008 25480887

[34] Zabotti A Zandonella Callegher S Gandolfo S Valent F Giovannini I Cavallaro E. Hyperechoic bands detected by salivary gland ultrasonography are related to salivary impairment in established Sjögren's syndrome. Clin Exp Rheumatol. (2019) 37 Suppl 118:146–52. 31365337

[35] Luciano N Baldini C Tarantini G Ferro F Sernissi F Varanini V. Ultrasonography of major salivary glands: a highly specific tool for distinguishing primary Sjögren's syndrome from undifferentiated connective tissue diseases. Rheumatology (Oxford). (2015) 54:2198–204. 10.1093/rheumatology/kev253 26206346

[36] Singh AG Singh S Matteson EL. Rate, risk factors and causes of mortality in patients with Sjögren's syndrome: a systematic review and meta-analysis of cohort studies. Rheumatology (Oxford). (2016) 55:450–60. 10.1093/rheumatology/kev354 26412810 PMC5009445

[37] Brito-Zerón P Kostov B Solans R Fraile G Suárez-Cuervo C Casanovas A. Systemic activity and mortality in primary Sjögren syndrome: predicting survival using the EULAR-SS Disease Activity Index (ESSDAI) in 1045 patients. Ann Rheum Dis. (2016) 75:348–55. 10.1136/annrheumdis-2014-206418 25433020

[38] Quartuccio L Isola M Baldini C Priori R Bartoloni Bocci E Carubbi F. Biomarkers of lymphoma in Sjögren's syndrome and evaluation of the lymphoma risk in prelymphomatous conditions: results of a multicenter study. J Autoimmun. (2014) 51:75–80. 10.1016/j.jaut.2013.10.002 24231556

[39] Retamozo S Brito-Zerón P Ramos-Casals M. Prognostic markers of lymphoma development in primary Sjögren syndrome. Lupus. (2019) 28:923–36. 10.1177/0961203319857132 31215845

[40] Quartuccio L Baldini C Bartoloni E Priori R Carubbi F Corazza L. Anti-SSA/SSB-negative Sjögren's syndrome shows a lower prevalence of lymphoproliferative manifestations, and a lower risk of lymphoma evolution. Autoimmun Rev. (2015) 14:1019–22. 10.1016/j.autrev.2015.07.002 26162302

[41] Romão VC Talarico R Scirè CA Vieira A Alexander T Baldini C. Sjögren's syndrome: state of the art on clinical practice guidelines. RMD Open. (2018) 4(Suppl. 1):e000789. 10.1136/rmdopen-2018-000789 30402274 PMC6203093

[42] Baer AN Grader-Beck T Antiochos B Birnbaum J Fradin JM. Ultrasound-guided biopsy of suspected salivary gland lymphoma in Sjögren's syndrome. Arthritis Care Res. (2020). 10.1002/acr.24203. [Epub ahead of print]. 32248649 PMC7541433

[43] Zabotti A Zandonella Callegher S Lorenzon M Pegolo E Scott CA Tel A. Ultrasound-guided core needle biopsy compared to open biopsy: a new diagnostic approach to major salivary gland enlargement in Sjögren's syndrome. Rheumatology (Oxford). (2020) keaa441. 10.1093/rheumatology/keaa441 32940706

[44] De Vita S Gandolfo S Zandonella Callegher S Zabotti A Quartuccio L. The evaluation of disease activity in Sjögren's syndrome based on the degree of MALT involvement: glandular swelling and cryoglobulinaemia compared to ESSDAI in a cohort study. Clin Exp Rheumatol. (2018) 36(Suppl. 112):150–6. 30156548

[45] Milic VD Petrovic RR Boricic IV Radunovic GL Pejnovic NN Soldatovic I. Major salivary gland sonography in Sjögren's syndrome: diagnostic value of a novel ultrasonography score (0–12) for parenchymal inhomogeneity. Scand J Rheumatol. (2010) 39:160–6. 10.3109/03009740903270623 20059370

[46] Hocevar A Ambrozic A Rozman B Kveder T Tomsic M. Ultrasonographic changes of major salivary glands in primary Sjogren's syndrome. Diagnostic value of a novel scoring system. Rheumatology (Oxford). (2005) 44:768–72. 10.1093/rheumatology/keh588 15741192

[47] Salaffi F Argalia G Carotti M Giannini FB Palombi C. Salivary gland ultrasonography in the evaluation of primary Sjögren's syndrome. Comparison with minor salivary gland biopsy. J Rheumatol. (2000) 27:1229–36. 10813292

[48] Leehan KM Pezant NP Rasmussen A Grundahl K Moore JS Radfar L. Minor salivary gland fibrosis in Sjögren's syndrome is elevated, associated with focus score and not solely a consequence of aging. Clin Exp Rheumatol. (2018) 36(Suppl. 112):80–8. 29148407 PMC5913007

[49] Baldini C Luciano N Tarantini G Pascale R Sernissi F Mosca M. Salivary gland ultrasonography: a highly specific tool for the early diagnosis of primary Sjögren's syndrome. Arthritis Res Ther. (2015) 17:146. 10.1186/s13075-015-0657-7 26022533 PMC4461980

[50] Giovagnorio F Pace F Giorgi A. Sonography of lacrimal glands in Sjögren syndrome. J Ultrasound Med. (2000) 19:505–9. 10.7863/jum.2000.19.8.505 10944035

[51] Fisher BA Everett CC Rout J O'Dwyer JL Emery P Pitzalis C. Effect of rituximab on a salivary gland ultrasound score in primary Sjögren's syndrome: results of the TRACTISS randomised double-blind multicentre substudy. Ann Rheum Dis. (2018) 77:412–6. 10.1136/annrheumdis-2017-212268 29275334 PMC5867400

[52] Jousse-Joulin S Devauchelle-Pensec V Cornec D Marhadour T Bressollette L Gestin S. Brief report: ultrasonographic assessment of salivary gland response to rituximab in primary Sjögren's syndrome. Arthritis Rheumatol. (2015) 67:1623–8. 10.1002/art.39088 25708147

[53] Bowman SJ Everett CC O'Dwyer JL Emery P Pitzalis C Ng W-F. Randomized controlled trial of rituximab and cost-effectiveness analysis in treating fatigue and oral dryness in primary Sjögren's syndrome. Arthritis Rheumatol. (2017) 69:1440–50. 10.1002/art.40093 28296257

[54] Pijpe J Kalk WWI van der Wal JE Vissink A Kluin PhM Roodenburg JLN. Parotid gland biopsy compared with labial biopsy in the diagnosis of patients with primary Sjogren's syndrome. Rheumatology. (2006) 46:335–41. 10.1093/rheumatology/kel266 16891656

[55] Mossel E Delli K van Nimwegen JF Stel AJ Kroese FGM Spijkervet FKL. Ultrasonography of major salivary glands compared with parotid and labial gland biopsy and classification criteria in patients with clinically suspected primary Sjögren's syndrome. Ann Rheum Dis. (2017) 76:1883–9. 10.1136/annrheumdis-2017-211250 28754802

