# Supplementary material for: Normal-Appearing Salivary Gland Ultrasonography Identifies a Milder Phenotype of Primary Sjögren's Syndrome
Source: Front Med (Lausanne). 2020 Dec 9;7:602354. doi: 10.3389/fmed.2020.602354 (PMC7756091; doi:10.3389/fmed.2020.602354)
Supplement: Supplementary file 1 [file Table_1.DOCX]

**Supplementary figure S1.** Agreement between the salivary gland ultrasound (SGUS) and the minor salivary gland biopsy (MSGB).


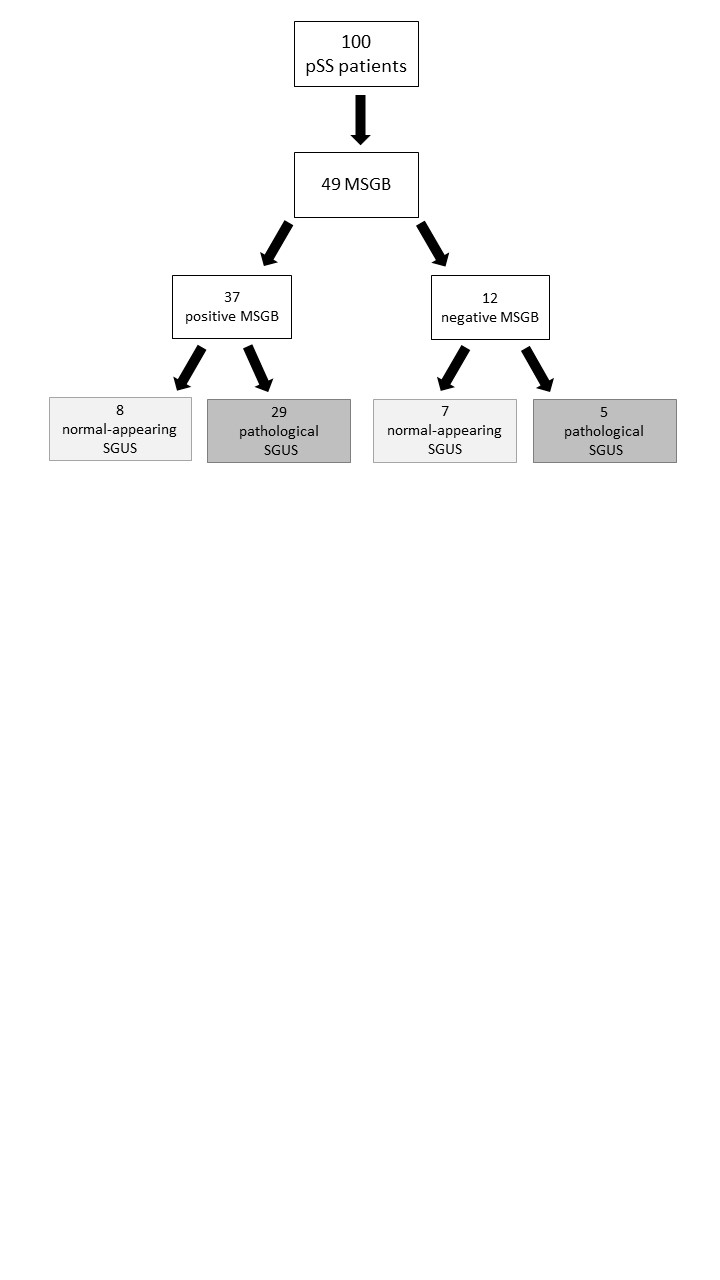


**Supplementary table S1.** ESSDAI domains in the all cohort and in the two groups of patients.

| **ESSDAI domains** | **All patients** | **SGUS**  **Normal-appearing group** | **SGUS**  **pathological group** | **p-value**  **normal *vs***  **pathological** |
| --- | --- | --- | --- | --- |
| Constitutional, *n (%)* | 0 | 0 | 0 | 1.000 |
| Lymphadenopathy, *n (%)* | 8/100 (8%) | 2/29 (6.9%) | 6/71 (8.4%) | 0.771 |
| Glandular, *n (%)* | 20/100 (20%) | 2/29 (6.9%) | 18/71 (25.3%) | **0.033** |
| Articular, *n (%)* | 19/100 (19%) | 7/29 (24.1%) | 12/71 (16.9%) | 0.500 |
| Cutaneous, *n (%)* | 7/100 (7%) | 1/29 (3.4%) | 6/71 (8.4%) | 0.372 |
| Pulmonary, *n (%)* | 3/100 (3%) | 0 | 3/71 (4.2%) | 0.263 |
| Renal, *n (%)* | 2/100 (2%) | 0 | 2/71 (2.8%) | 0.364 |
| Muscular, *n (%)* | 1/100 (1%) | 0 | 1/71 (1.4%) | 0.523 |
| Peripheral nervous system, *n (%)* | 10/100 (10%) | 3/29 (10.3%) | 7/71 (9.8%) | 0.953 |
| Central nervous system, *n (%)* | 1/100 (1%) | 1/29 (3.4%) | 0 | 0.118 |
| Haematological, *n (%)* | 33/100 (33%) | 6/29 (20.7%) | 27/71 (38%) | 0.082 |
| Biological, *n (%)* | 55/100 (55%) | 11/29 (37.9%) | 44/71 (62%) | **0.048** |

ESSDAI=EULAR Sjögren's Syndrome Disease Activity Index.
